# Incentive Mechanism and Subsidy Design for Construction and Demolition Waste Recycling under Information Asymmetry with Reciprocal Behaviors

**DOI:** 10.3390/ijerph17124346

**Published:** 2020-06-17

**Authors:** Peiyang Su, Ying Peng, Qidan Hu, Ruwen Tan

**Affiliations:** College of Architecture and Environment, Sichuan University, Chengdu 610065, China; supeiyang@stu.scu.edu.cn (P.S.); pengying@scu.edu.cn (Y.P.); 2017223050038@stu.scu.edu.cn (Q.H.)

**Keywords:** construction and demolition waste, information asymmetry, incentive mechanism, reciprocity

## Abstract

To solve information asymmetry, we adopted the principal-agent framework to design the incentive mechanisms between the remanufacturer and the collector in the construction and demolition (C&D) waste-recycling industry. By using the model of reciprocity, we analyzed how the entities’ behavioral motives affect their decisions in terms of the incentive mechanisms. The findings showed that the collector responds to their perception of the remanufacturer’s intentions. If the perception is positive, they will make more effort in the collection work. If not, less effort will be put forth. Most importantly, we found that reciprocity helps to save the remanufacturer cost in the incentive mechanisms and makes the collector choose a higher effort level in the collection work. This finding showed that reciprocity serves to solve information asymmetry. By conducting a numerical simulation, we found that although a high subsidy policy can achieve rapid improvement of recycling-supply-chain performance, it is inefficient in maintaining friendly cooperation between the remanufacturer and the collector.

## 1. Introduction

With the acceleration of urbanization, a large amount of construction and demolition (C&D) waste is generated every year, making the construction sector a major contributor to the growing environmental problems [1]. According to the “Report on the development of China’s construction waste recycling industry” released in 2014, approximately 1.6 to 2.4 billion tons of C&D waste are generated in China every year, and it is estimated that the annual output of C&D waste will exceed 7 billion tons by 2030. According to the data available, in 2014, almost 333 million tons of C&D waste were produced in Europe [2]; meanwhile, there were about 534 million tons (U.S. short tons) of C&D waste generated in the United States [3]. Moreover, it is estimated that a large portion of C&D waste is sent to landfills directly. For example, in the USA, approximately 30% of C&D waste is disposed of by landfilling [4]. More than half of the C&D waste in UK and about one-third of that in Australia is sent to landfills [5,6]. However, according to the Waste Framework Directive 2008/98/EC (WFD) approved by the European Commission, the last suggested waste-management option is “landfill”, which is considered to bring about many environmental problems, such as the emission of greenhouse gas, waste of land resources, contamination of groundwater or soil, etc. [7,8,9]. To manage C&D waste appropriately, an effective management strategy is urgently needed.

The importance of 3R principles (i.e., reduction, reuse, and recycling) in C&D waste management is illustrated in existing research [10,11]. With the application of 3R principles in the construction industry, C&D waste gradually becomes a valuable resource. Specifically, recycling is thought to be effective in utilizing the values of C&D waste and minimizing its environmental impacts [12,13]. However, the recycling and reuse rate of the C&D waste in China is less than 5% [11], indicating that the remaining C&D waste is arbitrarily buried or illegally dumped. In some European countries, including Greece, Portugal, Hungary, and Spain, the C&D waste recycling rates are less than 15% [1]. Therefore, how to increase the recycling rate of C&D waste is an important issue to be explored worldwide [14].

Existing studies have investigated the economic factors (e.g., the cost of recycling the C&D waste, governmental subsidy, etc.) leading to a decline in the C&D waste recycling rate [11,14,15,16,17]. It has been pointed out that whether project stakeholders decide to implement proper C&D waste management mainly depends on the costs they incur [14,15,16]. However, due to the high cost of the recycling management, including the gate fees of recycling plants, the C&D waste delivery cost, etc., it is not attractive for the entities to engage in the recycling activities [16,17]. Additionally, a lack of economic support (e.g., a proper subsidy policy provided for the recycling entities) is also considered to make recycling companies unable to sustain stable and profitable businesses, and it is suggested that more attention should be paid to proper economic incentives to prompt the development of the C&D waste recycling industry [11]. 

In general, the C&D waste recycling practice in some regions (e.g., mainland China) is still in its early stage. Due to lack of mature market mechanisms, there usually exists information asymmetry between the C&D waste recycling entities in these regions [17,18]. According to the literature [19,20,21], information asymmetry is common in the C&D waste recycling industry, and the asymmetrical distribution of information among stakeholders causes a decline in the efficiency of resource allocation. Because of information asymmetry, a large amount of C&D waste is sent to landfills instead of being properly collected and sorted by the professional business entities, leaving the C&D waste recycling plants with insufficient processed materials, which affects their production of recycled products [12].

In practice, the problem of information asymmetry in the C&D waste recycling industry can be described as follows. The remanufacturer entrusts a professional collector to collect C&D waste, which should be sorted and processed before being delivered to the remanufacturer. However, the remanufacturer cannot observe the collector’s effort level in the collection process, where “effort” here refers to the improvement of the collection channels, innovation of the collecting technology, upgrades of the collecting equipment, etc. As the remanufacturer does not have the adequate information about the collector’s effort level, when the output of the collection process is low, such as a low quantity of delivered C&D waste or low quality of the processed materials, the collector, who does not make reasonable effort, can attribute it to the unstable supply of or the low quality of the raw C&D waste in the market, which does not depend on its effort level. Moreover, this collector can still claim that sufficient investment has been made in the collection work and demand a “reasonable payoff” from the remanufacturer, improving their own profit, while the C&D waste recycling rate and the remanufacturer’s profit decrease. Such a phenomenon is called moral hazard due to information asymmetry in the C&D waste recycling industry. As proposed in the existing literature, the principal-agent theory can be used to analyze information asymmetry between business entities [22]. In a principal-agent framework, some entities (the principals) hire others (the agents) to provide services for them, and the agents’ payoffs depend on the quantity and quality of the provided services. To solve information asymmetry, our study designed effective incentive mechanisms using a principal-agent framework to encourage the collector, who is information-superior, to make reasonable effort [23,24]. When designing incentive mechanisms, it is crucial that the collector’s payoff be determined by the output of the collection process, and this strategy has been adopted by many remanufacturers in practice.

In addition to moral hazard, the collector’s behavioral motives beyond profit maximization also lower their effort level in the collection work. With an increase in the potential highest payoff the remanufacturer can pay for the collection work, a fair-minded collector usually demands a higher payoff that they think is fair. If this demand is not satisfied by the remanufacturer, there will be an observable decline in the quantity or quality of the delivered C&D waste materials, even though the collector’s payoff is reduced because of their poor performance in the collection work. Additionally, it has been found that employed agents respond to the principals’ intentions about how to share the potential final surplus, and the principals offering wages that are higher or potentially fairer will make their agents reciprocate with more effort, even if the increased investment cost lowers the agents’ profits [25]. In these two examples, it can be observed that when making an effort choice, the collector (or the agent) does not consider their profits to be optimal and responds to the intentions of the remanufacturer (or the principal) concerning fairness.

In the field of behavioral economics, laboratory and field experiments have been conducted to confirm the existence of behavioral motives that are beyond profit maximization in individuals’ decision-making [25,26]. To explain entities’ behaviors, the existing literature proposes the theory of “intention-based reciprocity” [27]. This concept assumes that a player cares about his opponent’s intentions. If he feels his opponent is kind to him, he will be nice to his opponent as well. If he feels that his opponent’s intentions are unkind, he will also be unkind to his opponent. This theory refers to decision-makers’ intentions, which can be used to reasonably explain the behavioral motives of the collector and the agent in the given examples. According to the hypothesis of “reciprocity”, the collector (the agent) compares their payoff with what they think is fair before making an effort choice. If the payoff offered by the remanufacturer (the principal) does not meet the collector’s expectation, the collector will perceive the remanufacturer’s intentions as negative and lower their effort level as a response. If the payoff meets the collector’s expectation, a positive perception of the remanufacturer’s intentions will be held and more effort will be put forth. To analyze the changes in the choices of the reciprocal remanufacturer and the reciprocal collector, we considered the entities’ preferences for reciprocity when designing incentive mechanisms.

Moreover, due to the fact that it is unlikely that the efficient allocation of resources will be achieved through the self-regulation of the recycling market, the government is suggested to play a role in the improvement of the recycling industry [28]. Generally, remanufacturers in the C&D waste recycling industry are mainly supported by the government’s subsidy policy, which is considered to play a significant role in the development of the C&D waste recycling industry [29]. However, it has been found that subsidy policies for the recycling industry are not always effective, and it has been argued whether and how the government should subsidize the entities in the recycling industry [17,30,31]. Specifically, Jia et al. (2017) pointed out that the single-subsidy policy has certain limitations, because a subsidy above a certain threshold makes the amount of recycled C&D waste decrease [15]. Therefore, it is important to explore the effects of the government’s subsidy policy on the C&D waste recycling performance (defined as the remanufacturer’s utility and the C&D waste recycling rate) when considering “reciprocity” within a principal-agent framework.

(1) These given examples offered motivation for our study. We aimed to answer the following questions. How does reciprocity affect the choices of the remanufacturer and the collector, and is it efficient in solving information asymmetry between these two entities?
(2) What effect does the government’s subsidy policy have on the performance of the recycling supply chain?

To solve the problem of information asymmetry, we used the principal-agent framework to design incentive mechanisms between the remanufacturer and the collector. By incorporating “reciprocity” into the designed incentive mechanisms, we derived the changes in the choices of these two entities and discussed whether reciprocity is efficient in solving information asymmetry between them. We used the model of “intention-based reciprocity” to formalize the entities’ reciprocal behaviors. By conducting a numerical simulation, we explored the effects of the government’s subsidy policy on the performance of the recycling supply chain.

Our study contributes to the literature on C&D waste recycling management from three perspectives. First, by using the theory of reciprocity, our findings explain collectors’ decisions to increase or decrease their effort level, which are influenced by their perception of the remanufacturer’s intentions. Second, we found that reciprocity helps to save the remanufacturers cost in the incentive mechanisms and makes the collector put forward more effort in their collection work. This finding showed that reciprocity serves to solve information asymmetry between these two entities. Third, we observed that a high subsidy policy for the remanufacturer induced negative perception from the collector of their opponent, which harms the friendly cooperation between them. The rest of our study is arranged as follows. Section 2 provides a review of the related literature. Section 3 presents the basic notations and assumptions of the incentive model. Section 4 explains the design of the incentive mechanism in the rational supply chain. Section 5 shows the development of the incentive mechanism by incorporating reciprocity. A numerical simulation was conducted to explore the effects of the subsidy policy and is reported in Section 6. The conclusions and management implications are given in Section 7.

## 2. Literature Review

We reviewed the related literature considering three aspects: C&D waste recycling management, the design of incentive mechanisms under information asymmetry, and behavioral motives.

### 2.1. C&D Waste Recycling Management

Existing literature on C&D waste recycling can be classified into two categories. The first one encompasses economic study of C&D waste recycling. For example, Zhao et al. (2010) evaluated the economic feasibility of C&D waste recycling in Chongqing, and they found that operating a C&D waste recycling center might represent a high investment risk [32]. Silva et al. (2017) found that the recycling approach is more beneficial than conventional demolition and disposal methods in C&D waste management, from both economic and environmental perspectives [16]. Other studies have also evaluated the economic performance of C&D waste recycling, and the findings have shown that C&D waste recycling achieves good economic performance [16,33].

The other category of literature includes studies of the environmental impacts of recycling C&D waste. Ortiz et al. (2010) evaluated the environmental impacts of C&D waste, and they found that in terms of global warming potential, the recycling approach is more environmentally friendly compared with incineration and landfilling [13]. Simion et al. (2013) analyzed the environmental impacts of landfilling and recycling C&D waste, and found that the environmental impacts of recycling C&D waste are 40% less than those of landfill [34]. Marzouk et al. (2014) also found that recycling C&D waste leads to lower emissions, energy use, and global warming potential, and conserves landfill space compared to landfilling [35].

These studies showed that the recycling method is beneficial to both the economic and environmental performance of C&D waste management. However, none of these studies considered information asymmetry in the C&D waste recycling industry, which our study aimed to solve by designing effective incentive mechanisms.

### 2.2. The Design of Incentive Mechanisms under Information Asymmetry

In the process of designing a contract or executing a contract, there exists some information that some entities know while the others do not, and such information is called “private information”. The existence of private information makes the distribution of information asymmetrical among the entities, which is “information asymmetry”.

Our study relates to literature on the principal-agent theory, which is usually used in the design of incentive mechanisms. According to Gailmard et al. (2014), the principal-agent theory encapsulates a rational choice modeling where the principal uses whatever actions to provide incentives for the agent to make decisions that the principal most prefers [36]. By testing principal-agent theory in buyer–supplier relationships, Steinle et al. (2014) found that both ex ante and ex post forms of opportunism can explain the occurrence of moral hazard [37]. Laffont and Martimort (2013) proposed the theory of incentive based on the principal-agent framework, and stated that the need for proper incentive mechanisms appears when the principal hires an agent who has superior information [21]. Cecchini et al. (2013) found that the principal-agent theory helped to analyze the effects caused by unobservable actions and external uncertainties, and that it provides an important framework for models with factors such as incentive mechanism design, information, and the performance of self-interested players [38]. Macho-Stadler and Pérez-Castrillo (2015) also stated that the principal-agent theory can identify the difficulties that arise from asymmetrical information distribution between two players and be adopted to design effective incentive mechanisms between them [39]. These studies showed the efficiency of the principal-agent theory in explaining moral hazard and designing incentive mechanisms to solve information asymmetry.

Our study is related to the literature on the design of incentive mechanisms in a supply-chain system. Xu et al. (2012) studied a dual information asymmetry problem between a manufacturer and a recycler. In their designed incentive mechanisms, the recycler had to invest their personal funds in the collection work, and they found that the more the recycler invested, the more effort they were willing to make, which helped to resolve the moral hazard [40]. Zhang et al. (2016) designed incentive mechanisms for the manufacturer to reduce the efficiency loss of a supply-chain system under information asymmetry, and they also verified the validation of the designed incentive mechanisms [41]. Khanjari et al. (2014) also designed incentive mechanisms for a manufacturer to induce their hired sales agent to exert optimal effort in a supply-chain system [42]. The difference between these studies and ours is that we considered the behavioral motives of the supply-chain members in their decision-making, which these studies did not consider.

### 2.3. Behavioral Motives

The existing literature illustrates that entities’ behavioral motives affect their decisions, which is beyond the assumption that material self-interests exclusively motivate decision-makers [27,43,44,45,46]. Anand and Goyal (2019) found that individuals’ behavioral motives are prevalent, which are not inconsequential deviations from self-interest, and that they have significant effects on outcomes and performances [45].

Among the existing literature, some considers the decision-makers’ behavioral motives as “inequity aversion”. For example, Katok and Pavlov (2013) found that inequity aversion has the most explanatory power regarding retailers’ behaviors contributing to poor performance of supply-chain contracts [44]. Fehr and Schmidt (1999) proposed that one has more disutility when one’s earning is less than that of others (disadvantageous inequity), and one has less disutility when one’s earning is more than that of others (advantageous inequity) [26]. It was shown that, whether the earning is more or less than others, entities respond negatively to an unequal outcome in models of inequity aversion, which was not suitable for our study. The study of inequity aversion only cares about the equitable allocation of the material payoffs, which lacks consideration of the players’ underlying intentions.

Others have studied the effects of “reciprocity” on entities’ decision-making. Rabin (1993) defined “reciprocity” as “people like to help those who are helping them, and to hurt those who are hurting them”, and proposed a model to formalize decision-makers’ reciprocal behaviors [27]. In recent years, some literature has shown that the role of reciprocity is decisive, and that supply-chain members’ optimal decisions are affected by their reciprocal behaviors [46]. Du et al. (2014) studied how reciprocity affects supply-chain members’ decisions and the channel’s coordination between a supplier and a retailer. They found that the entities’ intentions play important roles in their decision-making [46]. In a behavioral study, Niederhoff and Kouvelis (2016) found that entities’ preferences for reciprocity exist, and that some of them are willing to sacrifice their profits to reach an equitable profit allocation, which was not as predicted by traditional profit-maximization analysis [43]. They also found that entities’ observed kind behaviors can help the system perform better, which supported the analytical predictions made in the study of Katok and Pavlov (2013) [44]. These studies show that the theory of reciprocity can be adopted to explain entities’ behavioral motives beyond profit maximization, and that their intentions play important roles in their decision-making.

We reviewed the literature on behavioral motives from two perspectives: “inequity aversion” and “reciprocity”. By comparing these two concepts, we found that studies of reciprocity care about entities’ intentions rather than merely the equitable allocation of material payoffs, which helped us to give a reasonable explanation of entities’ decisions beyond self-interest.

## 3. The Model

### 3.1. Model Description

**Figure 1:**
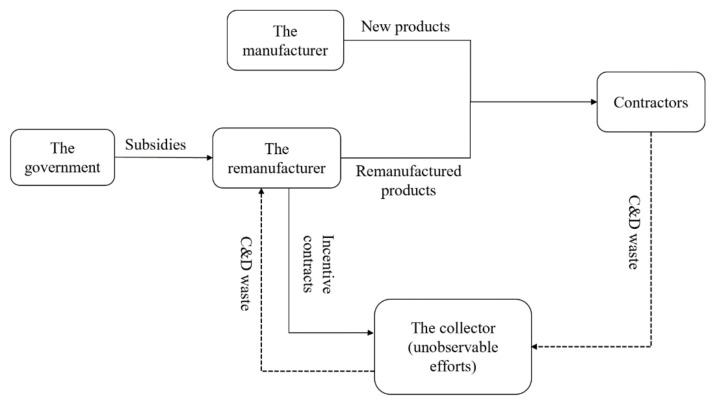
Process flow chart of the construction and demolition (C&D) waste recycling procedure.

As shown in Figure 1, we designed a closed C&D waste supply chain under the government’s subsidy, in which a manufacturer produces new construction products (hereinafter called “the new products”) using natural materials, and a remanufacturer entrusts the C&D waste collection work to a third-party collector and produces remanufactured construction products (hereinafter called “the remanufactured products”) using the collected C&D waste materials; both the new and remanufactured products are sold to the contractors. Specifically, a system comprising a remanufacturer and a collector was mainly studied. Within a principal-agent framework, the remanufacturer was seen as the principal, and the collector was seen as an agent.

**Figure 2:**
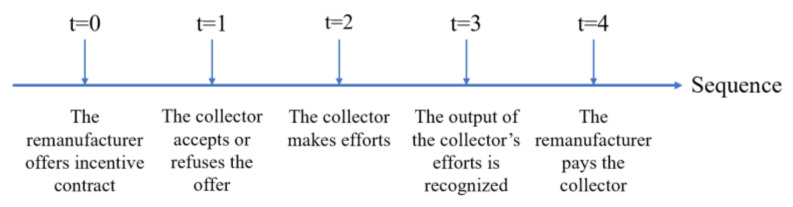
The sequence of the incentive mechanism.

When there is information asymmetry, the collector’s effort level remains unobservable except for by the collector, which generates a moral hazard problem. To solve the moral hazard problem, the remanufacturer offers an incentive mechanism to the collector to exert their optimal effort. The sequence of the incentive mechanism in our research is shown in Figure 2.

Moreover, to explore the effects of “reciprocity” on the choices of the entities, we built two models. The first one was called the “rational model”, where both the remanufacturer and the collector are rational and all they want is to maximize their own material utilities. The second one was called the “reciprocal model”, in which they both care about their opponents’ intentions and the entities’ decision-making is influenced by their perceptions of their opponents’ intentions.

### 3.2. Notations

In our study, a manufacturer, a remanufacturer, and a collector were considered, with subscript “*m*” representing the manufacturer, subscript “r” representing the remanufacturer, and subscript “c” representing the collector. Moreover, we use the superscripts “ * ” and “ ** ” to represent the variables or parameters in the rational and reciprocal models, respectively. The notations and definitions of the variables and the parameters used in our study are shown in Table 1.

### 3.3. Assumptions

To simplify the models, we proposed the assumptions below.

Assumption 1. Both the manufacturer and the remanufacturer are risk-neutral, and they aim to maximize their own expected utility, which is equivalent to their expected profit. Meanwhile, the collector is risk-averse, and the introduction of the collector’s risk aversion and its effects are given in the following.

Assumption 2. The C&D waste collection rate is linearly related to the collector’s effort level. In our study, all the collected C&D waste was used to produce the remanufactured products, meaning that the C&D waste recycling rate was equal to the C&D waste collection rate. The recycling rate is given by

$$\tau \left(a\right)=\varphi \;a,\;\varphi >0.$$

The hypothesis that the recycling rate (quantity) of the C&D waste is linearly correlated to the collector’s effort level was supported by existing studies [47,48]. Therefore, the quantity of recycled C&D waste is τM owned by the remanufacturer and is defined as $\pi_{e}=h\;a+\epsilon_{1}$ (h>0)  [49,50,51]. Here,$\;\epsilon_{1}$ represents the exogenous uncertainties (e.g., the unstable supply of or the low quality of the raw C&D waste in the market, which does not depend on the collector’s effort level) and follows a normal distribution, $\epsilon_{1}\sim N\left(0,\sigma^{2}\right)$, in which $\sigma^{2}$ represents the variance of uncertainty.

Assumption 3. For the contractor, there are two types of products to choose from—new products and remanufactured products. These two types of products are substitutes for each other, which means the demand for one type will be affected by the other one’s price. Moreover, the demands of both products are also influenced by their own prices [52]. Therefore, the demand functions are given by

$$D_{m}=\phi -bP_{m}+dP_{r},$$

$$D_{r}=\phi -bP_{r}+dP_{m}-\tau M.$$

where ϕ represents the basic market size. The proposed demand functions were supported by References [53,54], where b≥d>0 indicates that the effect of one type of product’s price is greater than the cross-price effect, and the quantity of recycled waste will cause a reduction in the demand for the remanufactured products.

### 3.4. The Formulation of Utility Functions

For the collector, the remanufacturer can only observe the gross output $\pi_{e}$ of collection work, and the collector is paid based on the value $\pi_{e}$. The collector’s payoff is

$$S\left(\pi_{e}\right)=\alpha +\beta \pi_{e},\;0\leq \beta \leq 1.$$

where  α represents a fixed payment paid by the remanufacturer, and β is the ”incentive level”, which indicates how much the collector’s payoff depends on the output $\pi_{e}$. The linear compensation rule of payoff $S\left(\pi_{e}\right)$ was supported by existing studies [50,55,56]. During the collection process, the collector incurs the cost of collection effort:$\;C\left(a\right)=-a^{2}k/2$, k>0. The quadratic cost function of the collection effort implies that the collector needs to invest more to raise their effort level, which is supported by existing studies [38,56]. To sum up, the collector’s expected profits consist of the payoff provided by the remanufacturer and the effort cost, which is

$$E\left(\omega \right)=E\left(S\left(\pi_{e}\right)-C\left(a\right)\right)=-a^{2}k/2+\alpha +\beta ah.$$

When accepting an incentive mechanism offered by the remanufacturer, the risk-averse collector usually does not choose a high “incentive level” β, because the higher the “incentive level” β, the more the collector’s payoff depends on the output $\pi_{e}$. However, the output $\pi_{e}$ is determined not only by the collector’s effort level, but also by the uncertainties in the market which do not depend on the collector’s effort level, for example, the unstable supply of the raw C&D waste in the area where the collector does its collection work. When such uncertainties occur, even though the collector has made effort as demanded, the poor output of the collection makes its payoff lower. Considering the collector’s risk-averse attitude, existing studies propose that the collector incurs a risk cost $\frac{1}{2}\rho var\left(\omega \right)=\frac{1}{2}\beta^{2}\rho \sigma^{2}\;(\rho >0)$ [48,57]. By taking the risk cost into the profit function $E\left(\omega \right)$, we derived the collector’s utility function as

$$E\left(U_{c}\right)=E\left(\omega \right)-\frac{1}{2}\beta^{2}\rho \sigma^{2}=-a^{2}k/2+\alpha +\beta ah-\frac{1}{2}\beta^{2}\rho \sigma^{2}$$

For the remanufacturer, the government offers subsidies to it for two purposes: to increase the C&D waste recycling rate (environmental factor) and to improve the remanufacturer’s utility (economic factor) [58]. The total subsidies offered to the remanufacturer depend on the output of the recycling process [59], which is

(1) T=δτM.

The remanufacturer’s utility consists of the output of collection, collector’s payoff, government’s subsidies, and net profits from selling the remanufactured products. The remanufacturer’s expected utility is

$$E\left(U_{r}\right)=ah\left(1-\beta \right)-\alpha +aM\delta \varphi +\left(-c_{r}+P_{r}\right)\left(-aM\varphi +\phi +dP_{m}-bP_{r}\right).$$

The manufacturer’s profit is

(2) $${\;}_{\;P_{m}\;}^{max}E\left(U_{m}\right)=\left(P_{m},-,c_{m}\right)\left(\phi -bP_{m}+dP_{r}\right).$$

It should be noted that the numbered equations are used to introduce the formulation or calculation process of solving the models in the Appendixes.

## 4. Incentive Model in a Rational Supply Chain

In this section, we built an incentive model using a principal-agent framework for the remanufacturer and the collector in a rational supply chain, wherein both the remanufacturer (principal) and the collector (agent) wish to maximize their own utility.

According to the general principal-agent framework, the principal’s objective to maximize his utility is constrained by the agent’s utility maximization. Thus, the incentive model is given by

$$\mathrm{maximize}\;E\left(U_{r}\right)$$

(3) $$s.t.\;\mathrm{maximize}\;E\left(U_{c}\right)$$

(4) $$E\left(U_{c}\right)\geq u_{0}$$

Constraint (3) is the constraint that gives the collector, who chooses the optimal effort level, the highest utility in the incentive mechanism. Constraint (4) is called the participation constraint, indicating that the collector will not accept the incentive contract unless it is at least offered a reservation utility (the agent’s lowest utility to accept this incentive mechanism). When solving the optimal strategy set, the participation constraint can be restricted to an equation [21].

In our study, the relationship between the remanufacturer and collector corresponded to the Stackelberg game theory model, in which the remanufacturer makes decisions first, and the collector will take actions considering the decisions made by the remanufacturer; meanwhile, the actions of the collector are supposed to have already been predicted by the remanufacturer, and, thus, the remanufacturer makes decisions taking the collector’s actions into account. Moreover, the models were solved using the backward induction method, and the calculation process is shown in the Appendixes.

**Proposition** **1.** In the rational model, the collector’s optimal effort level is $a^{*}={h\beta }^{*}/k$, and the optimal C&D waste recycling rate is $\tau^{*}\left(a^{*}\right)={\;\varphi h\beta }^{*}/k$.

Proposition 1 shows that, in a rational supply chain, the effort level of the collector and the C&D waste recycling rate are determined by the optimal “incentive level” $\beta^{*}$. When the optimal incentive level $\beta^{*}$ increases, the rational collector makes more effort, and, thus, the C&D waste recycling rate is also increased. The higher the incentive level $\beta^{*}$, the more the collector’s payoff depends on the output of collection work, which serves to induce the collector to exert more effort.

**Proposition** **2.** In the rational model, the optimal incentive mechanism set by the remanufacturer is $\left\{\alpha^{*},\beta^{*}\right\}$, and the expressions are $\alpha^{*}=\left(a^{*}{}^{2}k-2a^{*}h\beta^{*}+2u_{0}+\beta^{*}{}^{2}\rho \sigma^{2}\right)/2$, $\beta^{*}=\frac{hk\left(\left(2b+d\right)\left(-M\phi \varphi +2b\left(h+M\delta \varphi \right)-d\left(h+M\delta \varphi \right)\right)-bdM\varphi c_{m}+\left(2b^{2}-d^{2}\right)M\varphi c_{r}\right)}{k\left(4b^{2}-d^{2}\right)\left(h^{2}+k\rho \sigma^{2}\right)-2bh^{2}M^{2}\varphi^{2}}$.

Proposition 2 gives the optimal fixed payment $\alpha^{*}$ and the optimal incentive level $\beta^{*}$, which determine the collector’s payoff in a rational supply chain.

**Corollary** **1.** *In the rational model*,

(1) *When the amount of the C&D waste is below a certain threshold*$\left(M^{2}<M_{0}=\frac{\left(4b^{2}-d^{2}\right)k\left(h^{2}+k\rho \sigma^{2}\right)}{2bh^{2}\varphi^{2}}\right)$, *an increase in the unit subsidy*δ*for the remanufacturer raises the collector’s effort level and the C&D waste recycling rate*$\left(i.e.,\;\frac{\partial a^{*}}{\partial \delta }>0,\;\frac{\partial \tau^{*}}{\partial \delta }>0\right)$;
(2) *When the amount of the C&D waste is above a certain threshold*$\left(M^{2}>M_{0}=\frac{\left(4b^{2}-d^{2}\right)k\left(h^{2}+k\rho \sigma^{2}\right)}{2bh^{2}\varphi^{2}}\right)$ , *an increase in the unit subsidy* δ *for the remanufacturer lowers the collector’s effort level and the C&D waste recycling rate* $\left(i.e.,\;\frac{\partial a^{*}}{\partial \delta }<0,\;\frac{\partial \tau^{*}}{\partial \delta }<0\right)$.

Corollary 1 shows that, when the total amount of the C&D waste is below a certain threshold $\left(M^{2}<M_{0}\right)$, with the unit subsidy increasing, the collector’s effort level and the C&D waste recycling rate increase. However, if the amount of the C&D waste is above that threshold $\left(M^{2}>M_{0}\right)$, increasing the subsidy makes the collector’s effort level and the C&D waste recycling rate decrease.

## 5. Incentive Model in a Reciprocal Supply Chain

Compared with the rational model, we formulated an incentive model by considering the role of “reciprocity” (hereinafter called the “reciprocal model”).

### 5.1. Basic Formulation

In this section, we explain three crucial concepts of reciprocity proposed by Rabin (1993) [27], which are “material payoffs”, “kindness function”, and “the adjusted utility function”.

#### 5.1.1. Material Payoffs

Suppose there are two players (*i* and *j*) in one game, and both would reciprocate the other’s actions on the basis of their perception of each other. There are two strategy spaces $S_{i}$ and $S_{j}$, for Players *i* and *j*, respectively. When Player *j* chooses their action $b_{j}$, Player *i* chooses their action $a_{i}$ from their payoff space $\pi \left(b_{j}\right)≜\{\pi_{i}\left(a_{i},b_{j}\right)|a_{i}\in S_{i}\}$, and meanwhile, Player *i*’s material payoff is $\pi_{i}\left(a_{i},b_{j}\right)$.

When Player *j* chooses action $b_{j}$, their highest payoff is $\pi_{j}^{h}\left(b_{j}\right)$ and their lowest payoff is $\pi_{j}^{l}\left(b_{j}\right)$. It is assumed that Player *j*’s “equitable payoff” is defined as $\pi_{j}^{fair}\left(b_{j}\right)=\frac{\pi_{j}^{h}\left(b_{j}\right)+\pi_{j}^{l}\left(b_{j}\right)}{2}$, which is a reference point of how kind Player *i* appears.

#### 5.1.2. Kindness Function

According to Rabin (1993) and Fehr and Schmidt (2006) [27,60], the “kindness function” showing Player *i*’s kindness to Player j is defined as

(5) $$f_{i}\left(a_{i},b_{j}\right)=\frac{\pi_{j}\left(a_{i},b_{j}\right)-\pi_{j}^{fair}\left(b_{j}\right)}{\pi_{j}^{h}\left(b_{j}\right)-\pi_{j}^{l}\left(b_{j}\right)}$$

It should be noted that the action chosen by Player *i* affects the value of $f_{i}\left(a_{i},b_{j}\right)$. If $f_{i}\left(a_{i},b_{j}\right)>0$, Player *j* gets more payoff than the “equitable payoff”, and perceives Player *i* as “kind”; and if $f_{i}\left(a_{i},b_{j}\right)<0$, Player *j* perceives Player *i* as “unkind”.

Similarly, Player i’s belief in Player *j*’s kindness is given by

$${\tilde{f}}_{j}\left(b_{j},c_{i}\right)=\frac{\pi_{i}\left(b_{j},c_{i}\right)-\pi_{i}^{fair}\left(c_{i}\right)}{\pi_{j}^{h}\left(b_{j}\right)-\pi_{i}^{l}\left(c_{i}\right)}.$$

where $c_{i}$ represents Player *i*’s belief in which strategy Player *j* believes Player *i* chooses. ${\tilde{f}}_{j}\left(b_{j},c_{i}\right)$ is formally equivalent to $f_{j}\left(a_{i},b_{j}\right)$, which is the actual kindness Player *j* shows to Player *i*.

#### 5.1.3. The Adjusted Utility Function

The new expected utility $U_{i}\left(a_{i},b_{j},c_{i}\right)$ of Player *i* consists of both the material utility and players’ shared notion of reciprocity:

(6) $$\begin{array}{ll} U_{i} & \left(a_{i},b_{j},c_{i}\right)=\pi_{i}\left(a_{i},b_{j}\right)+{\tilde{f}}_{j}\left(b_{j},c_{i}\right)+{\tilde{f}}_{j}\left(b_{j},c_{i}\right)f_{i}\left(a_{i},b_{j}\right)=\pi_{i}\left(a_{i},b_{j}\right)+\\ & {\tilde{f}}_{j}\left(b_{j},c_{i}\right)\;\left[1+f_{i}\left(a_{i},b_{j}\right)\right]=\pi_{i}\left(a_{i},b_{j}\right)+f_{j}\left(a_{i},b_{j}\right)\left[1+f_{i}\left(a_{i},b_{j}\right)\right]. \end{array}$$

Apparently, Player *i* chooses to maximize their expected utility. The formulation of Player *j*’s new utility function is similar.

We used the model of reciprocity to formalize the entities’ perceptions of their opponent’s intentions. It was assumed that in the reciprocal model, the incentive level $\beta^{fair}=\beta^{*}$. Both the remanufacturer and the collector can adjust their opponent’s perception by varying their decisions $\alpha^{fair}$ and $a^{fair}$. The interval of $\alpha^{fair}$ and $a^{fair}$ are $\left[0,\bar{\alpha }\right]$ and $\left[0,\bar{a}\right]$, in which $\bar{\alpha }$ represents the highest fixed payment and $\bar{a}$ represents the collector’s highest effort level.

**Proposition** **3.**

(1) *The collector’s kindness perceived by the remanufacturer is*$f_{C}=\frac{2a^{fair}-\bar{a}}{2\bar{a}}$;
(2) *The remanufacturer’s kindness perceived by the collector is*$f_{R}=\frac{2\alpha^{fair}-\bar{\alpha }}{2\bar{\alpha }}$.

Proposition 3 indicates that one’s decision has effects on one’s kindness as perceived by the other one. For the remanufacturer, their kindness perceived by the collector is determined by the fixed payment $\alpha^{fair}$ and the payment the collector thinks is fair $\bar{\alpha }/2$. When the fixed payment offered by the remanufacturer is above the fair payment ($\alpha^{fair}\geq \bar{\alpha }/2$), their perceived kindness $f_{R}$ will be positive, which means that the remanufacturer is perceived as “kind” by the collector; however, when the fixed payment is below the fair payment ($\alpha^{fair}<\bar{\alpha }/2$), the remanufacturer is perceived as “unkind”. We found that, with a given fair payment $\bar{\alpha }/2$, the more actual fixed payment $\alpha^{fair}$ the remanufacturer offers to the collector, the more “kind” they are perceived by the collector. It was also found that the remanufacturer’s kindness as perceived by the collector depended not only on the actual fixed payment $\alpha^{fair}$ offered to the collector, but also on the fair payment $\bar{\alpha }/2$ that the collector thinks they should receive. If the remanufacturer offers equal fixed payment $\alpha^{fair}$ to two collectors with different fair payments $\bar{\alpha }/2$, the remanufacturer will be perceived as more “kind” by the collector who has a lower fair payment $\bar{\alpha }/2$.

### 5.2. Modeling of the Reciprocal Model

By incorporating reciprocity into the incentive model, the reciprocal model can be written as follows.

$$\mathrm{maximize}\;E\left(U_{r}^{fair}\right)\;s.t.\;\mathrm{maximize}\;E\left(U_{c}^{fair}\right)\;E\left(U_{c}^{fair}\right)\geq u_{0}.$$

The solution to this incentive model is similar to the rational model, and by solving this model, the incentive mechanism considering reciprocity can be derived.

**Proposition** **4.** *In the reciprocal model*,

(1) *The collector’s optimal effort level is*$a^{**}=\frac{2\alpha^{**}-\bar{\alpha }+2\bar{a}h\bar{\alpha }\beta^{*}}{2k\bar{a}\bar{\alpha }}$;
(2) *The fixed payment is*$\alpha^{**}=a^{**}{}^{2}k/2-\beta^{*}ah+\beta^{*}{}^{2}\rho \sigma^{2}/2-\left(2a^{**}+\bar{a}\right)\left(2\alpha^{**}-\bar{\alpha }\right)/\left(4\bar{a}\bar{\alpha }\right)+u_{0}$;
(3) *The price of the remanufactured products is*$P_{r}{}^{**}=\frac{2b\phi +d\phi +\bar{a}bM\varphi +2\bar{a}bM\bar{\alpha }\varphi +bdc_{m}+2b^{2}c_{r}-\frac{bM\varphi \sqrt{-4\bar{\alpha }+k{\left(\bar{a}+2\bar{a}\bar{\alpha }\right)}^{2}+4\bar{a}h\left(1+2\bar{\alpha }\right)\beta^{*}+4\beta^{*}{}^{2}\rho \sigma^{2}+8u_{0}}}{\sqrt{k}}}{4b^{2}-d^{2}}$;
(4) *The price of the new products is*$P_{m}{}^{**}=\frac{4b\phi +2d\phi +\bar{a}dM\varphi +2\bar{a}dM\bar{\alpha }\varphi +4b^{2}c_{m}+2bdc_{r}-\frac{dM\varphi \sqrt{-4\bar{\alpha }+k{\left(\bar{a}+2\bar{a}\bar{\alpha }\right)}^{2}+4\bar{a}h\left(1+2\bar{\alpha }\right)\beta^{*}+4\beta^{*}{}^{2}\rho \sigma^{2}+8u_{0}}}{\sqrt{k}}}{8b^{2}-2d^{2}}$.

Proposition 4 shows how the entities in a reciprocal supply chain will adjust their decisions compared with those in a rational supply chain, which is analyzed in the following sections.

**Lemma** **1.**

(1) *If the remanufacturer offers a fixed payment*$\alpha^{*}$higher than the threshold$\bar{\alpha }/2$*in a rational supply chain, they will be perceived as “kind” by the collector*$\left(\alpha^{**}>\bar{\alpha }/2\right)$*in a reciprocal supply chain*.
(2) *If the remanufacturer offers fixed payment*$\alpha^{*}$lower than the threshold$\bar{\alpha }/2$*in a rational supply chain, they will be perceived as “unkind” by the collector*$\left(\alpha^{**}<\bar{\alpha }/2\right)$*in a reciprocal supply chain*.

The findings of Lemma 1 indicated that by comparing the fixed payment $\alpha^{*}$, which is decided by the rational remanufacturer, with the collector’s fair payment $\bar{\alpha }/2$, we can tell whether the remanufacturer would be perceived as “kind” by the collector in the reciprocal supply chain. If the rational remanufacturer offers a fixed payment higher than the fair payment $\left(\alpha^{*}>\bar{\alpha }/2\right)$, they will be perceived as “kind” in the reciprocal supply chain ($\alpha^{**}>\bar{\alpha }/2$); if the fixed payment offered by the rational remanufacturer is lower than the fair payment $\left(\alpha^{*}<\bar{\alpha }/2\right)$, the remanufacturer will be perceived as “unkind” in the reciprocal supply chain ($\alpha^{**}<\bar{\alpha }/2$).

We simplified the process of identifying the collector’s perception of the remanufacturer. Next, we discuss how the collector’s choices are influenced by their perception of the remanufacturer.

**Corollary** **2.** *In a reciprocal model, for the collectors*,

(1) *If they perceive the remanufacturer as “kind”* ( $\alpha^{**}>\bar{\alpha }/2$ *)*, *their effort level will be raised* $\left(a^{**}>a^{*}\right)$ *and the recycling rate will increase* $\left(\tau^{**}>\tau^{*}\right)$.
(2) *If they perceive the remanufacturer as “unkind”* ( $\alpha^{**}<\bar{\alpha }/2$ ) *their effort level will be lowered* $\left(a^{**}<a^{*}\right)$, *and the recycling rate will decrease* $\left(\tau^{**}<\tau^{*}\right)$.

Corollary 2 indicates that when the remanufacturer is perceived as “kind” ($\alpha^{**}>\bar{\alpha }/2$), the collector will put more effort into the C&D waste collection work, and the recycling rate will increase. However, when the remanufacturer is perceived as “unkind” ($\alpha^{**}<\bar{\alpha }/2$), the collector will put in less effort, and thus, the C&D waste recycling rate will decrease. From Corollary 2, we found that the reciprocal collector’s perception of the remanufacturer has effects on their choices. A positive perception will encourage the collector to put forth more effort, which is conductive to the improvement of recycling rate, while a negative perception will discourage the collector, which makes the recycling rate decrease.

**Corollary** **3.** *In the reciprocal model, for the remanufacturers*,

(1) *If they are perceived as “kind” by the collector*$\left(\alpha^{**}>\bar{\alpha }/2\right)$ , *the fixed payment in a reciprocal supply chain will be lower than that in a rational supply chain* $\left(\alpha^{**}<\alpha^{*}\right)$.
(2) *If they are perceived as “unkind” by the collector*$\left(\alpha^{**}<\bar{\alpha }/2\right)$ , *the fixed payment in a reciprocal supply chain will be higher than that in a rational supply chain* $\left(\alpha^{**}>\alpha^{*}\right)$.

Corollary 3 shows that if the remanufacturer is perceived as “kind” by the collector $\left(\alpha^{**}>\bar{\alpha }/2\right)$, the fixed payment paid to the collector will be lower than that in a rational supply chain, and if the remanufacturer is perceived as “unkind” $\left(\alpha^{**}<\bar{\alpha }/2\right)$, then the fixed payment will be higher. Associated with Proposition 3 and Corollary 2, we found that when the collector perceives the remanufacturer as “kind”, even though the fixed payment is lower than that in a rational supply chain, the collector will choose a higher (compared with the decision made in a rational supply chain) effort level as a response to this positive perception; however, when the remanufacturer is perceived as “unkind”, even though the fixed payment is higher than that in a rational supply chain, the collector will choose a lower effort level as a response to the negative perception. The above findings show that the reciprocal collector responds to their perception of the remanufacturer when making decisions. Moreover, the collector’s decision-making depends not only on how much fixed payment $\alpha^{**}$ the remanufacturer actually offers, but also on the payment $\bar{\alpha }/2$, which is perceived as fair.

Next, we explored how the new and remanufactured products’ prices change when the collector’s perception differs.

**Corollary** **4.** *In a reciprocal model*,

(1) *If the fixed payment*$\alpha^{**}>\bar{\alpha }/2$ , *the price of the remanufactured products* $P_{r}{}^{**}<P_{r}{}^{*}$ *will hold; and if the fixed payment* $\alpha^{**}<\bar{\alpha }/2$ , *the price of the remanufactured products* $P_{r}{}^{**}>P_{r}{}^{*}$ *will hold*;
(2) *If the fixed payment*$\alpha^{**}>\bar{\alpha }/2$ , *the price of the new products* $P_{m}{}^{**}<P_{m}{}^{*}$ *will hold; and if the fixed payment* $\alpha^{**}<\bar{\alpha }/2$ , *the price of the new products* $P_{m}{}^{**}>P_{m}{}^{*}$ *will hold*.

Corollary 4 shows that if the remanufacturer is perceived as “kind” by the collector, the price of the remanufactured products will be lowered in a reciprocal supply chain. Because of the “price competition” between the new and remanufactured products, the manufacturer will also choose to lower the price of the new products. If the remanufacturer is perceived as “unkind”, both products’ prices will be raised.

**Corollary** **5.** *In a reciprocal model*,

(1) *If the collector perceives the remanufacturer as “kind”* ( $\alpha^{**}>\bar{\alpha }/2$ ), *the collector’s total payoff in a reciprocal supply chain will be lower than that in a rational supply chain*$\left(S^{**}<S^{*}\right)$.
(2) *If the collector perceives the remanufacturer as “unkind”* ($\alpha^{**}<\bar{\alpha }/2$) *the collector’s total payoff in a reciprocal supply chain will be higher than that in a rational supply chain* $\left(S^{**}>S^{*}\right)$.

From Corollary 5, it was observed that when the collector’s perception of the remanufacturer is positive ($\alpha^{**}>\bar{\alpha }/2$), the collector receives a lower total payoff than that in rational supply chain; when the perception is negative $\left(\alpha^{**}>\bar{\alpha }/2\right)$, the collector receives a higher total payoff than that in rational supply chain. We also find that when the collector perceives the remanufacturer as “kind”, even though the remanufacturer offers lower total payoff, the collector will make more effort to reciprocate the remanufacturer’s kindness, and thus the recycling rate will be improved. However, when the collector’s perception of the remanufacturer is “unkind”, even though the remanufacturer offers higher total payoff, the collector will make less effort, which makes the recycling rate decrease. These findings are similar to the findings in Corollary 3.

The above findings proved collectors’ preference for reciprocity when making decisions, and such reciprocal behaviors can be explained as follows.

A positive perception can be understood as a positive signal received by the collector that the remanufacturer is willing to sustain friendly cooperation between two entities, which makes up for the reduction in the collector’s material payoff. To respond to the remanufacturer’s “kindness”, the collector chooses to make more effort in their collection work. In contrast, a negative perception lowers the collector’s expectations of their cooperation. Even though the remanufacturer offers higher payoff for the collector, this cannot make up the loss of the collector’s psychological utility. Therefore, the collector chooses to make less effort. Clearly, there is a trade-off for the collector between material payoff and psychological utility, which is influenced by perception of the remanufacturer. It can also be concluded that the collector’s positive perception not only lowers the remanufacturer’s incentive cost, but also makes the collector put forth more effort, which shows that “reciprocity” is efficient in solving information asymmetry.

We analyzed both entities’ decisions in a reciprocal supply chain. Next, we explored the effects of the government’s subsidy on the performance of the recycling supply chain.

**Corollary** **6.** *In a reciprocal model*,

(1) *When the amount of the C&D waste is below a certain threshold*$\left(M^{2}<M_{0}=\frac{\left(4b^{2}-d^{2}\right)k\left(h^{2}+k\rho \sigma^{2}\right)}{2bh^{2}\varphi^{2}}\right)$ , *an increase in the unit subsidy* δ *raises the collector’s effort level and the C&D waste recycling rate* $\left(i.e.,\;\frac{\partial a^{**}}{\partial \delta }>0,\;\frac{\partial \tau^{**}}{\partial \delta }>0\right)$.
(2) *When the amount of the C&D waste is above a certain threshold*$\left(M^{2}>M_{0}=\frac{\left(4b^{2}-d^{2}\right)k\left(h^{2}+k\rho \sigma^{2}\right)}{2bh^{2}\varphi^{2}}\right)$ , *an increase in the unit subsidy* δ *lowers the collector’s effort level and the C&D waste recycling rate* $\left(i.e.,\;\frac{\partial a^{**}}{\partial \delta }<0,\;\frac{\partial \tau^{**}}{\partial \delta }<0\right)$.

Corollary 6 shows that if the total amount of the C&D waste is below a certain threshold $\left(M^{2}<M_{0}\right)$, a higher unit subsidy will raise the collector’s effort level and the C&D waste recycling rate; however, if not $\left(M^{2}>M_{0}\right)$, a higher unit subsidy will lower the collector’s effort level and the C&D waste recycling rate, which indicates that a subsidy policy for the remanufacturer is not always suggested. It is also worth noting that compared with Corollary 1, the thresholds ($M_{0}=\frac{\left(4b^{2}-d^{2}\right)k\left(h^{2}+k\rho \sigma^{2}\right)}{2bh^{2}\varphi^{2}}$) in both the rational and the reciprocal models were equal, around which the effects of the subsidy policy on the performance of a recycling supply chain were different. The finding showed that the threshold $M_{0}$ existed in both models and was not influenced by the consideration of “reciprocity”. To simplify, we called factors affecting the effects of the subsidy policy on the recycling rate, which are not influenced by the entities’ behavioral motives, the “objective factors” (e.g., the total amount of the C&D waste in our study). When designing a subsidy policy, the government needs to take these “objective factors” into account.

## 6. Numerical Simulation

To conduct a numerical simulation, an appropriate value for each parameter should be assigned. At present, recycled bricks have the widest range of applications in China among all the C&D waste recycled products [59]. Therefore, we use recycled bricks as an example for our simulation. Data used in this numerical simulation were obtained from two channels. The first was a literature review. For example, we obtained the collector’s cost for the C&D waste collection work from Reference [9]. The other channel was a survey we conducted in a C&D waste recycling plant in Sichuan, China. In the conducted survey, we held interviews with the owner and a manager of the recycling plant to collect data such as the collector’s revenue from selling raw C&D waste materials, the production cost of the recycled bricks, etc. The units, values, and sources of the data are shown in Table 2.

It should be noted that RMB is the legal currency of China, and 1 RMB is approximately equal to 0.14 US dollars.

Suppose that the total amount of the C&D waste is sufficient to produce 50 blocks of the recycled bricks in our numerical simulation, and the demand for bricks (new and recycled) is 100 blocks. Associated with Table 2, we assigned values to the parameters as: M=5 (×10 blocks), ϕ=10 (×10 blocks), $c_{m}=2$ (RMB/10 blocks), $c_{r}=1.5$ (RMB/10 blocks), $\delta \in \left[0.25,1\right]$ (RMB/10 blocks). The remaining parameters were mainly determined by the collector’s specific characteristics, which were $\bar{a}=3$, h=1, k=0.5, φ=0.2, ρ=1, $\sigma^{2}=0.2$, b=4, d=2.

With the collector’s revenue from selling the raw waste materials and the cost of the collection work, we checked whether the parameter values were consistent with the collected data. When δ=1, it was obtained from the theoretical model that the collector’s revenue from selling the raw materials was $\alpha^{*}=2.24$ and the cost of the collection work was $C_{0}=0.931$. Moreover, the recycling rate was $\tau^{*}=0.368$. Therefore, when the total amount of the C&D waste was sufficient to produce 50 blocks of the recycled bricks, the raw materials sent to the remanufacturer could be used to produce 0.368×50=18.4 blocks of the recycled bricks. With each block of the recycled brick weighing 3.30 kg, the total weight of the delivered bricks was 60.72 kg. Therefore, the collector’s revenue from selling these raw C&D waste materials was $2.24\times \left(1000/60.72\right)=36.89$ RMB/tonne and the cost of the collection work was $0.931\times \left(1000/60.72\right)=15.33$ RMB/tonne, which were consistent with the data in Table 2.

Next, we used δ=1 as an example to analyze how the collector’s perception of the remanufacturer influences the remanufacturer’ utility.

**Figure 3:**
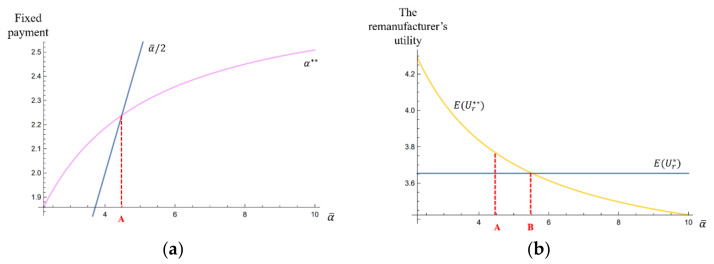
The effects of the highest fixed payment $\bar{\alpha }$ on (**a**) the fixed payment $\alpha^{**}$, and (**b**) the remanufacturer’s utilities $E\left(U_{r}^{*}\right)$ and $E\left(U_{r}^{**}\right)$ (when the unit subsidy δ=1 ).

Figure 3a depicts the curve of the fixed payment $\alpha^{**}$, taking the highest fixed payment $\bar{\alpha }$ as the varying parameter.

Specifically, on the left side of the threshold A, the remanufacturer is perceived as “kind” by the collector; while on the right side of A, the remanufacturer is perceived as “unkind”. We found that with the increase of the fair payment $\bar{\alpha }/2$, the remanufacturer’s kindness perceived by the collector was lower. This is because with the collector’s fair payment $\bar{\alpha }/2$ increasing, even though the collector receives more fixed payment $\alpha^{**}$, the difference between the fair payment $\bar{\alpha }/2$ and the actual fixed payment $\alpha^{**}$ is bigger, which makes the remanufacturer be perceived as less “kind”.

Figure 3b depicts the curves of the remanufacturer’s utilities $E\left(U_{r}^{*}\right)$ and $E\left(U_{r}^{**}\right)$, taking the highest fixed payment $\bar{\alpha }$ as the varying parameter.

Specifically, on the left side of the threshold A, $E\left(U_{r}^{**}\right)>E\left(U_{r}^{*}\right)$ holds. On the right side of A, when $A<\bar{\alpha }<B$, $E\left(U_{r}^{**}\right)>E\left(U_{r}^{*}\right)$; and when $\bar{\alpha }>B$, $E\left(U_{r}^{**}\right)<E\left(U_{r}^{*}\right)$. This observation illustrated that if the unit subsidy δ is sufficiently high, the utility of the remanufacturer, who is perceived as “kind” or “slightly unkind”, will be higher than that in the rational supply chain; however, when the remanufacturer is perceived as “strictly unkind”, the utility will be lower than that in the rational supply chain. These observations showed that, even though the collector might choose to lower their effort level to reciprocate their negative perception, a high subsidy can still improve the utility of an “unkind” remanufacturer. As a result, it makes the remanufacturer be satisfied with decisions that are not good for sustaining friendly cooperation with the collector.

**Figure 4:**
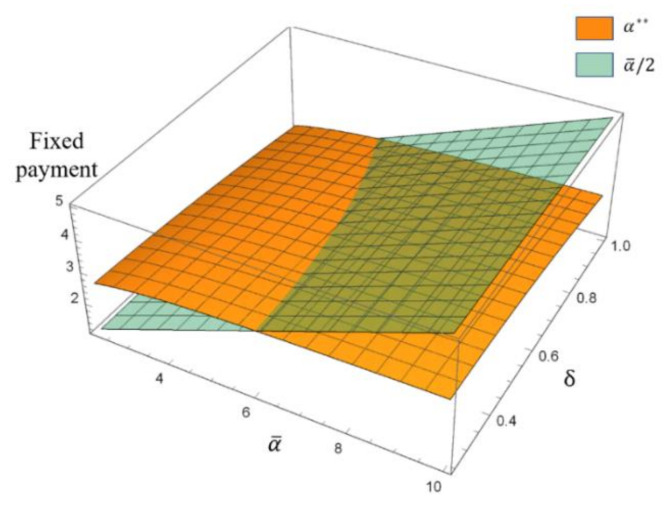
The effects of the highest fixed payment $\bar{\alpha }$ and the unit subsidy δ on the fixed payment $\alpha^{**}$.

Figure 4 shows the effects of the highest fixed payment $\bar{\alpha }$ and the unit subsidy δ on the fixed payment $\alpha^{**}$.

As shown in Figure 4, with a given highest fixed payment $\bar{\alpha }$, a higher unit subsidy δ led to lower fixed payment $\alpha^{**}$. Moreover, according to Lemma 1, on the left side of the line where two surfaces intersect (hereafter called the “perception boundary”), the remanufacturer is perceived as “kind”, while on the right side of the “perception boundary”, the remanufacturer is perceived as “unkind”. Moreover, we noticed that with an increasing unit subsidy, the collector’s perception of the remanufacturer changed from “positive” to “negative” in certain regions. This finding was consistent with the observation in Figure 3b that a sufficiently high subsidy for the remanufacturer is not good for friendly cooperation among the entities in the recycling industry.

Although it was found that a sufficiently high subsidy cannot maintain friendly cooperation between remanufacturer and collector, the government may adopt a higher subsidy policy to achieve a rapid improvement of the C&D waste recycling rate.

**Figure 5:**
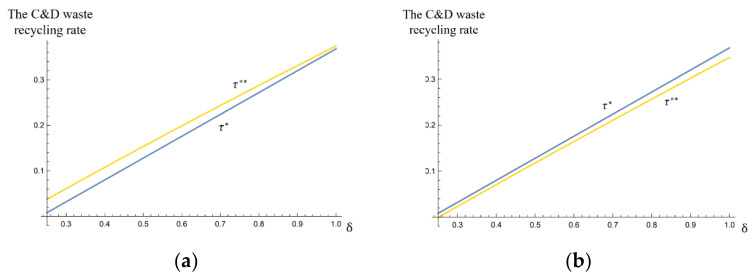
The effects of the unit subsidy δ on the recycling rates $\tau^{*}$ and $\tau^{**}$ (**a**) with the highest fixed payment $\bar{\alpha }=4$, and (**b**) with the highest fixed payment $\bar{\alpha }=7$.

We used $\bar{\alpha }=4$ and $\bar{\alpha }=7$ as examples to analyze the effects of the unit subsidy δ on the C&D waste recycling rates $\tau^{*}$ and $\tau^{**}$, as shown in Figure 5.

When the remanufacturer was perceived as “unkind” (i.e., $\bar{\alpha }=7$), $\tau^{**}<\tau^{*}$ held, and a higher unit subsidy δ led to a bigger difference between the recycling rates $\tau^{*}$ and $\tau^{**}$. When the remanufacturer was perceived as “kind” (i.e., $\bar{\alpha }=4$), the recycling rate $\tau^{**}>\tau^{*}$ held, but a higher unit subsidy δ reduced the marginal recycling rate. We found that even though a higher subsidy policy increased the C&D waste recycling rate, it led to a decline in the marginal recycling rate. This is because with a higher unit subsidy, the collector perceives the remanufacturer as less “kind”, and decides to lower their effort level in response. The effects of a lower effort level (reducing the recycling rate) offset the effects of a high subsidy policy (improving the recycling rate). This finding revealed that when designing a subsidy policy for remanufacturers, there is a trade-off for the government between achieving a rapid improvement of the supply-chain performance and maintaining friendly cooperation between the remanufacturer and the collector.

In the previous sections, we explained the advantages and disadvantages of a high subsidy policy. Next, we explored the effects of a low subsidy policy.

We used $\bar{\alpha }=4$ and $\bar{\alpha }=7$ as examples to analyze the effects of the unit subsidy δ on the remanufacturer’s utilities $E\left(U_{r}^{*}\right)$ and $E\left(U_{r}^{**}\right)$.

**Figure 6:**
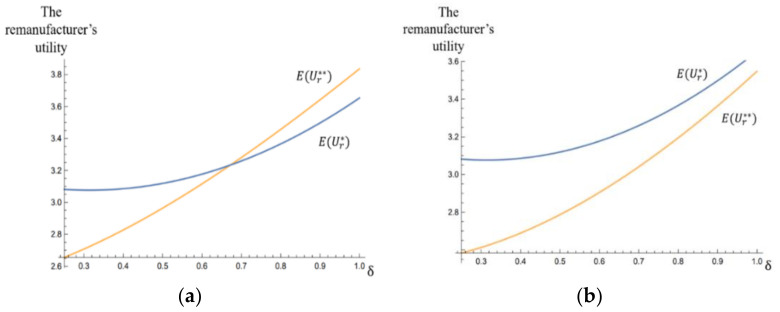
The effects of the unit subsidy δ on the remanufacturer’s utilities $E\left(U_{r}^{*}\right)$ and $E\left(U_{r}^{**}\right)$ (**a**) with the highest fixed payment $\bar{\alpha }=4$, and (**b**) with the highest fixed payment $\bar{\alpha }=7$.

From Figure 6a,b, it can be observed that the remanufacturer’s utilities $E\left(U_{r}^{*}\right)$ and $E\left(U_{r}^{**}\right)$ increased with the unit subsidy δ. With a sufficiently low unit subsidy, the remanufacturer’s utility was lower than that in the rational supply chain. We also found that when the remanufacturer was perceived as “unkind” ($\bar{\alpha }=7$), its utility could not be improved compared with that in the rational supply chain through a high subsidy policy, but when it was perceived as “kind” ($\bar{\alpha }=4$), a sufficiently high subsidy made its utility higher than that in the rational supply chain. These observations showed that a sufficiently low subsidy is not good for the remanufacturer’s utility, and a high subsidy policy helps improve the utility of a “kind” remanufacturer.

The findings showed that a low subsidy policy is not conducive to improvements of the remanufacturer’s utility and the C&D waste recycling rate, and, although a high subsidy policy is conducive to the improvement of recycling rate, it is not good for the friendly cooperation between the remanufacturer and the collector.

Next, we explored the effects of a reasonable subsidy policy.

**Figure 7:**
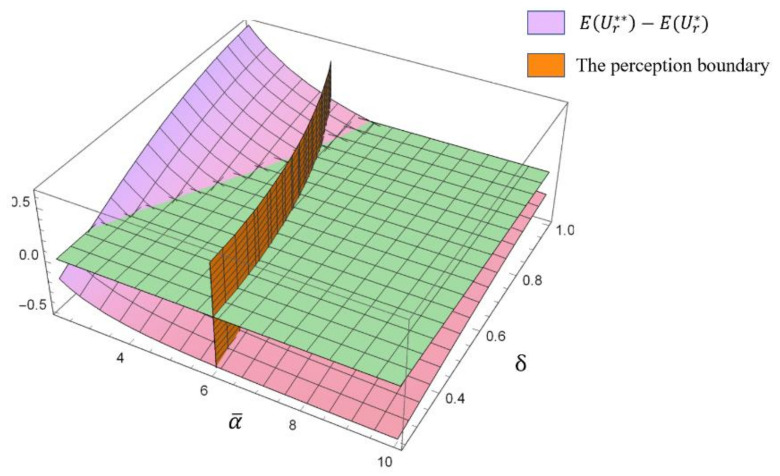
The effects of the highest fixed payment $\bar{\alpha }$ and the unit subsidy δ on the difference between the remanufacturer’s utilities $E\left(U_{r}^{**}\right)-E\left(U_{r}^{*}\right)$.

The “perception boundary” is shown in Figure 7. On the left side of the boundary, the remanufacturer is perceived as “kind”, while on the right side of the boundary, the remanufacturer is perceived as “unkind”.

From Figure 7, it can be observed that on the right side of the “perception boundary”, where the remanufacturer was perceived as “unkind”, there existed regions where $E\left(U_{r}^{**}\right)-E\left(U_{r}^{*}\right)>0$ (with a sufficiently high unit subsidy δ), which refers to the finding that a sufficiently high subsidy policy made the remanufacturer be satisfied with “unkind” decisions perceived by the collector. As a result, the friendly cooperation between the remanufacturer and the collector could not be maintained. On the left side of the “perception boundary”, there existed regions where $E\left(U_{r}^{**}\right)-E\left(U_{r}^{*}\right)<0$ (with a sufficiently low unit subsidy δ), which referred to the finding that a sufficiently low subsidy policy was not beneficial to the remanufacturer’s utility. Therefore, a proper subsidy range is shown in Figure 7, where an “unkind” remanufacturer’s utility is lower than that in the rational supply chain, and a “kind” remanufacturer’s utility can be improved through a reasonable subsidy policy (with the unit subsidy $\delta \in \left[0.36,\;0.86\right]$). The proposed subsidy policy helps to improve the remanufacturer’s utility and maintains friendly cooperation between the remanufacturer and the collector.

## 7. Conclusions

To solve information asymmetry between the remanufacturer and the collector in the C&D waste recycling industry, we designed incentive mechanisms using a principal-agent framework. We also used the model of “intention-based reciprocity” to formalize the behavioral motives are beyond profit maximization in individuals’ decision-making. This enabled us to find out how “reciprocity” affected the choices of the remanufacturer and the collector in the designed incentive mechanisms, and their efficiency in solving information asymmetry. Furthermore, by conducting a numerical simulation, we explored the effects of the government’s subsidy policy on the performance of the recycling supply chain. The main findings of our study were as follows.

Our study quantified the entities’ perceptions of their opponent’s intentions in a reciprocal supply chain, which helped us to derive the reciprocal collector’s behavior pattern in the designed incentive mechanisms: the collector’s decision-making depends not only on the actual payoff they receive, but also on the payoff they think is fair. By comparing the actual payoff they receive with the fair payoff they think they should get, the collector forms their perception of the remanufacturer’s intentions. When the collector perceives the remanufacturer as “kind”, they put forth more effort. When they perceive the remanufacturer as “unkind”, less effort is put forth.

Our findings showed how “reciprocity” affects the choices of the remanufacturer and the collector in designed incentive mechanisms. When the remanufacturer was perceived as “kind” by the collector, even though the collector’s payoff was lower than that in the rational supply chain, the collector made more effort than they did in the rational supply chain to reciprocate the remanufacturer’s “kind” intentions. When the remanufacturer was perceived as “unkind” by the collector, even though the collector’s payoff was higher than that in the rational supply chain, they chose to make less effort as a response to the opponent’s “unkind” intentions. Most importantly, we found that “reciprocity” helps to save the remanufacturer’s cost on the incentive mechanisms and makes the collector put more effort into their collection work. This finding showed that “reciprocity” is efficient in solving information asymmetry between the remanufacturer and the collector.

We also explore the effects of the government subsidy on the supply-chain performance. We found that when there was a large amount of C&D waste, an increase in the unit subsidy lowered the collector’s effort level, and the recycling rate also decreased. Therefore, a subsidy policy for the remanufacturer is not always suggested. Furthermore, we found that although a sufficiently high subsidy was efficient in achieving rapid improvements of the remanufacturer’s utility and the C&D waste recycling rate, it also made the remanufacturer be satisfied with “unkind” (as perceived by the collector) decisions, and made the collector have a more negative perception of the remanufacturer’s intentions. These two effects indicated that a high subsidy policy has negative effects on maintaining friendly cooperation between the remanufacturer and the collector.

Moreover, we have some suggestions for remanufacturers and policymakers in the C&D waste recycling industry. For the remanufacturers, it is important to ensure that the collectors have positive perceptions of them, because this not only helps to save the remanufacturers costs on incentive mechanisms, but also makes the collectors put forth more effort. For policymakers, when implementing a high subsidy policy, there is a trade-off between a rapid improvement of the supply-chain performance and the friendly cooperation among the entities in the C&D waste recycling industry.

This study obtained some interesting findings about information asymmetry and entities’ reciprocity in the C&D waste recycling industry. However, there are also important factors we did not consider, such as the quality of the recycled products. In practice, it is crucial to establish a quality-control system in the production of the recycled products. Additionally, we only considered moral hazard, which is just one type of information asymmetry. The consideration of dual information asymmetry could help to improve the application of our model. The data used in our numerical simulation were not enough. More available data are needed to verify and improve the applicability of the proposed model in practice. Moreover, our research could be expanded by considering multi-stage cooperation of the remanufacturer and the collector, which captures the presence of the C&D waste recycling industry. Furthermore, only the subsidy policy was analyzed in our study, which means that the role of the government was insufficiently explored, and more policies for C&D waste management need be explored in future studies.

## Figures and Tables

**Table 1:** Notations and definitions of the decision variables and the parameters.

Notations	Definitions
Decision variables	
α	Fixed payment
β	Incentive level
$P_{m},P_{r}$	Prices of the new products and the remanufactured products
a	The collector’s effort level in the construction and demolition (C&D) waste collection work
Parameters	
b	Price elasticity coefficient of the product’s demand function
d	Substitution coefficient between the new products and the remanufactured products, b≥d>0
$c_{m},c_{r}$	Unit production cost of the new products and the remanufactured products, $c_{r}<c_{m}$, $c_{r}\leq P_{r}$, $c_{m}\leq P_{m}$
φ	Effect coefficient of the collector’s effort level
M	Total amount of the C&D waste
k	Cost coefficient of the collector’s effort level
ρ	Risk aversion coefficient of the collector, ρ>0
δ	The unit subsidy for the recycled C&D waste

**Table 2:** Data of the numerical simulation.

Data	Value	Unit	Data Sources
Subsidy to the recycling plants	0.02	RMB/block	[59]
	0.04		[59]
	0.10		Survey
Production cost of the new bricks	0.20	RMB/block	Survey
Production cost of the recycled bricks	0.15	RMB/block	[59] and survey
Weight of the recycled bricks	3.30	kg/block	[61] and survey
The collector’s revenue from selling the raw construction and demolition (C&D) waste materials	35–45	RMB/tonne	Survey
The collector’s cost for the collection work	15	RMB/tonne	[9]

## Appendix A

**Proof of Proposition** **1.** From $\partial^{2}E\left(U_{c}\right)\;/\partial a^{2}=-a<0$, we find that collector’s expected utility function is concave in a. Using the first-order condition $\partial E\left(U_{c}\right)/\partial a=0$, we find the optimal effort level $a^{*}=h\beta /k$. The C&D waste recycling rate is expressed as $\tau^{*}\left(a^{*}\right)=\varphi a^{*}=\varphi h\beta /k$. □

## Appendix B

**Proof of Proposition** **2.** The origin objective function and constraints can be converted as:

(A1) $${\;}_{\;\alpha ,\beta ,P_{r}\;}^{\;max}E\left(U_{r}\right)=ah\left(1-\beta \right)-\alpha +aM\delta \varphi +\left(c_{r}-P_{r}\right)\left(aM\varphi -\phi -dP_{m}+bP_{r}\right)$$

(A2) $$s.t.\;a^{*}=h\beta /k$$

(A3) $$-a^{2}k/2+\alpha +\beta ah-\beta^{2}\rho \sigma^{2}/2=u_{0}$$

From (A3), we get

(A4) $$\alpha =\left(a^{2}k-2ah\beta +2u_{0}+\beta^{2}\rho \sigma^{2}\right)/2$$

We used the backward induction method to solve this model. By substituting (A2) and (A4) into (A1), the remanufacturer’s utility function is converted as

(A5) $${\;}_{\;\beta ,P_{r}\;}^{\;max}E\left(U_{r}\right)=-\frac{h^{2}\left(-1+\beta \right)\beta }{k}+\frac{\beta^{2}\left(h^{2}-k\rho \sigma^{2}\right)}{2k}+\frac{hM\beta \delta \varphi }{k}+\left(c_{r}-P_{r}\right)\left(-\phi +\frac{hM\beta \varphi }{k}-dP_{m}+bP_{r}\right)-u_{0}$$

The Hessian matrix of the remanufacturer’s utility function is given as

(A6) $$H\;=\left(\begin{array}{cc} -2b & -\frac{hM\varphi }{k}\\ -\frac{hM\varphi }{k} & -\frac{h^{2}+k\rho \sigma^{2}}{k} \end{array}\right).$$

From (A6), conditions of joint concavity of $E\left(U_{r}\right)$ are expressed as: (a)$\left|H_{1}\right|=-2b<0$; (b)$\;\left|H_{2}\right|>0$, which means $2bk\left(h^{2}+k\rho \sigma^{2}\right)-h^{2}M^{2}\varphi^{2}>0$. From first-order conditions $\partial E\left(U_{r}\right)/\partial \beta =0$ and $\partial E\left(U_{r}\right)/\partial P_{r}=0$, we get

(A3) $$\beta^{*}=\frac{h\left(h+M\delta \varphi +M\varphi c_{r}-M\varphi P_{r}\right)}{h^{2}+k\rho \sigma^{2}}$$

(A4) $$P_{r}{}^{*}=\frac{k\phi -hM\beta \varphi +bkc_{r}+dkP_{m}}{2bk}$$

Meanwhile, from the first-order condition of Equation (2), the new products’ price is

(A5) $$P_{m}{}^{*}=\frac{\phi +bc_{m}+dP_{r}}{2b}.$$

Considering the price competition between new and remanufactured products, we combine (A8), (A9) and get

(A6) $$P_{r}{}^{*}=-\frac{-2bk\phi -dk\phi +2bhM\beta \varphi -bdkc_{m}-2b^{2}kc_{r}}{\left(4b^{2}-d^{2}\right)k},$$

(A7) $$P_{m}{}^{*}=-\frac{-2bk\phi -dk\phi +dhM\beta \varphi -2b^{2}kc_{m}-bdkc_{r}}{\left(4b^{2}-d^{2}\right)k}.$$

By simultaneously solving (A7), (A10), and (A11), we get

$$\beta^{*}=\frac{hk\left(\left(2b+d\right)\left(-M\phi \varphi +\left(2b-d\right)\;\left(h+M\delta \varphi \right)\right)-bdM\varphi c_{m}+\left(2b^{2}-d^{2}\right)M\varphi c_{r}\right)}{k\left(4b^{2}-d^{2}\right)\left(h^{2}+k\rho \sigma^{2}\right)-2bh^{2}M^{2}\varphi^{2}},\;P_{r}{}^{*}=\frac{\begin{array}{c} 2bh^{2}k\phi +dh^{2}k\phi +2bk^{2}\rho \sigma^{2}\phi +dk^{2}\rho \sigma^{2}\phi -2bh^{3}M\varphi -2bh^{2}M^{2}\delta \varphi^{2}+bdk\left(h^{2}+k\rho \sigma^{2}\right)c_{m}\\ +2b\left(bk\left(h^{2}+k\rho \sigma^{2}\right)-h^{2}M^{2}\varphi^{2}\right)c_{r} \end{array}}{k\left(4b^{2}-d^{2}\right)\left(h^{2}+k\rho \sigma^{2}\right)-2bh^{2}M^{2}\varphi^{2}},\;P_{m}{}^{*}=\frac{\begin{array}{c} 2bh^{2}k\phi +dh^{2}k\phi +2bk^{2}\rho \sigma^{2}\phi +dk^{2}\rho \sigma^{2}\phi -dh^{3}M\varphi -dh^{2}M^{2}\delta \varphi^{2}-h^{2}M^{2}\phi \varphi^{2}+b\left(2bk\left(h^{2}+k\rho \sigma^{2}\right)-h^{2}M^{2}\varphi^{2}\right)c_{m}\\ +d\left(bk\left(h^{2}+k\rho \sigma^{2}\right)-h^{2}M^{2}\varphi^{2}\right)c_{r} \end{array}}{k\left(4b^{2}-d^{2}\right)\left(h^{2}+k\rho \sigma^{2}\right)-2bh^{2}M^{2}\varphi^{2}}.\;□$$

## Appendix C

**Proof of Corollary** **1.** When $M^{2}<\frac{\left(4b^{2}-d^{2}\right)k\left(h^{2}+k\rho \sigma^{2}\right)}{2bh^{2}\varphi^{2}}$, $\frac{\partial a^{*}}{\partial \delta }=\frac{\left(2b-d\right)\left(2b+d\right)h^{2}M\varphi }{\left(4b^{2}-d^{2}\right)k\left(h^{2}+k\rho \sigma^{2}\right)-2bh^{2}M^{2}\varphi^{2}}>0$, $\frac{\partial \tau^{*}}{\partial \delta }=\frac{\left(2b-d\right)\left(2b+d\right)h^{2}M\varphi^{2}}{\left(4b^{2}-d^{2}\right)k\left(h^{2}+k\rho \sigma^{2}\right)-2bh^{2}M^{2}\varphi^{2}}>0$. And when $M^{2}>\frac{\left(4b^{2}-d^{2}\right)k\left(h^{2}+k\rho \sigma^{2}\right)}{2bh^{2}\varphi^{2}}$, $\frac{\partial a^{*}}{\partial \delta }<0$, $\frac{\partial \tau^{*}}{\partial \delta }<0$. □

## Appendix D

**Proof of Proposition** **3.** By change the value of collector’s effort level α within $\left[0,\bar{\alpha }\right]$ from (A1), we can get the material utilities as follows

(A8) $$E{\left(U_{r}\right)}^{h}=-\alpha +\bar{a}h\left(1-\beta \right)+\bar{a}M\delta \varphi +\left(c_{r}-P_{r}\right)\left(-\phi +\bar{a}M\varphi -dP_{m}+bP_{r}\right),$$

(A9) $$E{\left(U_{r}\right)}^{l}=-\alpha +\left(c_{r}-P_{r}\right)\left(-\phi -dP_{m}+bP_{r}\right),$$

(A10) $$E{\left(U_{r}\right)}^{fair}=\frac{E{\left(U_{r}\right)}^{h}+E{\left(U_{r}\right)}^{l}}{2}=\frac{1}{2}\left(-2\alpha +\bar{a}h\left(1-\beta \right)+\bar{a}M\delta \varphi +\left(c_{r}-P_{r}\right)\left(-\phi -dP_{m}+bP_{r}\right)+\left(c_{r}-P_{r}\right)\left(-\phi +\bar{a}M\varphi -dP_{m}+bP_{r}\right)\right).$$

It should be noted that the inequation $E{\left(U_{r}\right)}^{h}\geq E{\left(U_{r}\right)}^{l}$ should be satisfied, which means $h\left(1-\beta \right)+M\delta \varphi +M\varphi \left(c_{r}-P_{r}\right)\geq 0$ holds. Further, we substitute (A12), (A13) and (A14) into Equation (5) and we get the remanufacturer’s perception of the collector’s “kindness” as

(A11) $$f_{C}=\frac{E\left(U_{r}\right)-E{\left(U_{r}\right)}^{fair}}{E{\left(U_{r}\right)}^{h}-E{\left(U_{r}\right)}^{l}}=\frac{2a-\bar{a}}{2\bar{a}}.$$

Similarly, the collector’s perception of the remanufacturer’s “kindness” is

(A12) $$f_{R}=\frac{E\left(U_{c}\right)-E{\left(U_{c}\right)}^{fair}}{E{\left(U_{c}\right)}^{h}-E{\left(U_{c}\right)}^{l}}=\frac{2\alpha -\bar{\alpha }}{2\bar{\alpha }}.\;□$$

## Appendix E

**Proof of Proposition** **4.** By incorporating (A15) and (A16) into Equation (6), we get

$$E\left(U_{r}^{fair}\right)=E\left(U_{r}\right)+f_{C}\left(1+f_{R}\right)=ah\left(1-\beta^{*}\right)-\alpha +aM\delta \varphi +\left(c_{r}-P_{r}\right)\left(aM\varphi -\phi -dP_{m}+bP_{r}\right)+\frac{\left(2a-\bar{a}\right)\left(2\alpha +\bar{\alpha }\right)}{4\bar{a}\bar{\alpha }}\;,$$

$$E(U_{c}^{fair})=E\left(U_{c}\right)+f_{R}\left(1+f_{C}\right)=-\frac{a^{2}k}{2}+\alpha +\beta^{*}ah-\frac{1}{2}\beta^{*}{}^{2}\rho \sigma^{2}+\frac{\left(2a+\bar{a}\right)\left(2\alpha -\bar{\alpha }\right)}{4\bar{a}\bar{\alpha }}.$$

From $\frac{\partial E(U_{c}^{fair})}{\partial a}=0$, we get collector’s effort level $a^{**}=\frac{2\alpha -\bar{\alpha }+2\bar{a}h\bar{\alpha }\beta^{*}}{2k\bar{a}\bar{\alpha }}=\frac{h\beta^{*}}{k}+\frac{2\alpha -\bar{\alpha }}{2k\bar{a}\bar{\alpha }}$; similarly to rational model, the incentive model can be converted as

(A17) $$\begin{array}{ll} {\;}_{\;\alpha ,\beta ,P_{r}\;}^{\;max}E\left(\;U_{r}^{fair}\right) & \\ & =ah\left(1-\beta^{*}\right)-\alpha +aM\delta \varphi +\left(c_{r}-P_{r}\right)\left(aM\varphi -\phi -dP_{m}+bP_{r}\right)\\ & +\frac{\left(2a-\bar{a}\right)\left(2\alpha +\bar{\alpha }\right)}{4\bar{a}\bar{\alpha }} \end{array}$$

(A18) $$s.t.\;a^{**}=\frac{2\alpha -\bar{\alpha }+2\bar{a}h\bar{\alpha }\beta^{*}}{2k\bar{a}\bar{\alpha }}$$

(A19) $$-\frac{a^{2}k}{2}+\alpha +\beta^{*}ah-\frac{1}{2}\beta^{*}{}^{2}\rho \sigma^{2}+\frac{\left(2a+\bar{a}\right)\left(2\alpha -\bar{\alpha }\right)}{4\bar{a}\bar{\alpha }}=u_{0}$$

Here, we used the backward induction method to solve this model. By incorporating (A18), (A19) into (A17), we get

(A20) $$E\left(U_{r}^{fair}\right)=\frac{1}{4{\bar{a}}^{2}k{\bar{\alpha }}^{2}}(2{\bar{a}}^{2}k{\bar{\alpha }}^{3}(-1+{\bar{a}}^{2}k+2{\bar{a}}^{2}k\bar{\alpha }+2\bar{a}h\beta^{*}-\bar{a}\sqrt{k}\sqrt{-4\bar{\alpha }+k{\left(\bar{a}+2\bar{a}\bar{\alpha }\right)}^{2}+4\bar{a}h\left(1+2\bar{\alpha }\right)\beta^{*}+4\beta^{*}{}^{2}\rho \sigma^{2}+8u_{0}})+2\bar{a}h\bar{\alpha }\left(-1+\beta^{*}\right)({\bar{a}}^{2}k\bar{\alpha }+2{\bar{a}}^{2}k{\bar{\alpha }}^{2}-\bar{a}\sqrt{k}\bar{\alpha }\sqrt{-4\bar{\alpha }+k{\left(\bar{a}+2\bar{\alpha }\bar{\alpha }\right)}^{2}+4\bar{a}h\left(1+2\bar{\alpha }\right)\beta^{*}+4\beta^{*}{}^{2}\rho \sigma^{2}+8u_{0}})-2\bar{a}M\bar{\alpha }\delta \varphi ({\bar{a}}^{2}k\bar{\alpha }+2{\bar{a}}^{2}k{\bar{\alpha }}^{2}-\bar{a}\sqrt{k}\bar{\alpha }\sqrt{-4\bar{\alpha }+k{\left(\bar{a}+2\bar{\alpha }\bar{\alpha }\right)}^{2}+4\bar{a}h\left(1+2\bar{\alpha }\right)\beta^{*}+4\beta^{*}{}^{2}\rho \sigma^{2}+8u_{0}})+\bar{\alpha }(2-{\bar{a}}^{2}k-2{\bar{a}}^{2}k\bar{\alpha }-2\bar{a}h\beta^{*}+\bar{a}\sqrt{k}\sqrt{-4\bar{\alpha }+k{\left(\bar{a}+2\bar{a}\bar{\alpha }\right)}^{2}+4\bar{a}h\left(1+2\bar{\alpha }\right)\beta^{*}+4\beta^{*}{}^{2}\rho \sigma^{2}+8u_{0}})(-2{\bar{a}}^{2}k\bar{\alpha }-2{\bar{a}}^{2}k{\bar{\alpha }}^{2}+\bar{a}\sqrt{k}\bar{\alpha }\sqrt{-4\bar{\alpha }+k{\left(\bar{a}+2\bar{a}\bar{\alpha }\right)}^{2}+4\bar{a}h\left(1+2\bar{\alpha }\right)\beta^{*}+4\beta^{*}{}^{2}\rho \sigma^{2}+8u_{0}})-2\bar{a}\bar{\alpha }\left(c_{r}-P_{r}\right)(2\bar{a}k\bar{\alpha }\phi +2\bar{a}dk\bar{\alpha }P_{m}-2\bar{a}bk\bar{\alpha }P_{r}+M\varphi ({\bar{a}}^{2}k\bar{\alpha }+2{\bar{a}}^{2}k{\bar{\alpha }}^{2}-\bar{a}\sqrt{k}\bar{\alpha }\sqrt{-4\bar{\alpha }+k{\left(\bar{a}+2\bar{a}\bar{\alpha }\right)}^{2}+4\bar{a}h\left(1+2\bar{\alpha }\right)\beta^{*}+4\beta^{*}{}^{2}\rho \sigma^{2}+8u_{0}})))$$

Taking the second-order partial derivative of (A20), we can get $\partial^{2}E\left(\;U_{r}^{fair}\right)/\partial P_{r}{}^{2}=-2b<0$, which shows that $E\left(\;U_{r}^{fair}\right)$ is concave in $P_{r}$. Thus, from $\partial E\left(\;U_{r}^{fair}\right)/\partial P_{r}=0$, we get

(A21) $$P_{r}{}^{fair}=\frac{2\phi +\bar{a}M\varphi +2\bar{a}M\bar{\alpha }\varphi +2bc_{r}+2dP_{m}-\frac{M\varphi \sqrt{-4\bar{\alpha }+k{\left(\bar{a}+2\bar{a}\bar{\alpha }\right)}^{2}+4\bar{a}h\left(1+2\bar{\alpha }\right)\beta^{*}+4\beta^{*}{}^{2}\rho \sigma^{2}+8u_{0}}}{\sqrt{k}}}{4b}$$

Considering the price competition between new and remanufactured products, we combine (A9), (A21) and get

(A22) $$P_{r}{}^{**}=\frac{2b\phi +d\phi +\bar{a}bM\varphi +2\bar{a}bM\bar{\alpha }\varphi +bdc_{m}+2b^{2}c_{r}-\frac{bM\varphi \sqrt{-4\bar{\alpha }+k{\left(\bar{a}+2\bar{a}\bar{\alpha }\right)}^{2}+4\bar{a}h\left(1+2\bar{\alpha }\right)\beta^{*}+4\beta^{*}{}^{2}\rho \sigma^{2}+8u_{0}}}{\sqrt{k}}}{4b^{2}-d^{2}},$$

(A23) $$P_{m}{}^{**}=\frac{4b\phi +2d\phi +\bar{a}dM\varphi +2\bar{a}dM\bar{\alpha }\varphi +4b^{2}c_{m}+2bdc_{r}-\frac{dM\varphi \sqrt{-4\bar{\alpha }+k{\left(\bar{a}+2\bar{a}\bar{\alpha }\right)}^{2}+4\bar{a}h\left(1+2\bar{\alpha }\right)\beta^{*}+4\beta^{*}{}^{2}\rho \sigma^{2}+8u_{0}}}{\sqrt{k}}}{8b^{2}-2d^{2}}.$$

By incorporating (A22) and (A23) into (A20), the remanufacturer’s expected utility function can be expressed as

$$E\left(U_{r}^{**}\right)=\frac{1}{4}(\frac{1}{\sqrt{k}}2h\left(-1+\beta^{*}\right)(\bar{a}\sqrt{k}+2\bar{a}\sqrt{k}\bar{\alpha }-$$

$$\sqrt{-4\bar{\alpha }+k{\left(\bar{a}+2\bar{a}\bar{\alpha }\right)}^{2}+4\bar{a}h\left(1+2\bar{\alpha }\right)\beta^{*}+4\beta^{*}{}^{2}\rho \sigma^{2}+8u_{0}})+$$

$$\frac{2M\delta \varphi \left(-\bar{a}\sqrt{k}-2\bar{a}\sqrt{k}\bar{\alpha }+\sqrt{-4\bar{\alpha }+k{\left(\bar{a}+2\bar{a}\bar{\alpha }\right)}^{2}+4\bar{a}h\left(1+2\bar{\alpha }\right)\beta^{*}+4\beta^{*}{}^{2}\rho \sigma^{2}+8u_{0}}\right)}{\sqrt{k}}+2\bar{\alpha }(-1+{\bar{a}}^{2}k+2{\bar{a}}^{2}k\bar{\alpha }+$$

$$2\bar{a}h\beta^{*}-\bar{a}\sqrt{k}\sqrt{-4\bar{\alpha }+k{\left(\bar{a}+2\bar{a}\bar{\alpha }\right)}^{2}+4\bar{a}h\left(1+2\bar{\alpha }\right)\beta^{*}+4\beta^{*}{}^{2}\rho \sigma^{2}+8u_{0}})+$$

$$\frac{1}{\bar{a}\sqrt{k}}\left(-2\bar{a}\sqrt{k}-2\bar{a}\sqrt{k}\bar{\alpha }+\sqrt{-4\bar{\alpha }+k{\left(\bar{a}+2\bar{a}\bar{\alpha }\right)}^{2}+4\bar{a}h\left(1+2\bar{\alpha }\right)\beta^{*}+4\beta^{*}{}^{2}\rho \sigma^{2}+8u_{0}}\right)(2-{\bar{a}}^{2}k-2{\bar{a}}^{2}k\bar{\alpha }-$$

$$2\bar{a}h\beta^{*}+\bar{a}\sqrt{k}\sqrt{-4\bar{\alpha }+k{\left(\bar{a}+2\bar{a}\bar{\alpha }\right)}^{2}+4\bar{a}h\left(1+2\bar{\alpha }\right)\beta^{*}+4\beta^{*}{}^{2}\rho \sigma^{2}+8u_{0}})+$$

$$\frac{4b}{{\left(-4b^{2}+d^{2}\right)}^{2}k}{\left(2b\sqrt{k}\phi +d\sqrt{k}\phi +\bar{a}b\sqrt{k}M\varphi +2\bar{a}b\sqrt{k}M\bar{\alpha }\varphi +bd\sqrt{k}c_{m}-2b^{2}\sqrt{k}c_{r}+d^{2}\sqrt{k}c_{r}-bM\varphi \sqrt{-4\bar{\alpha }+k{\left(\bar{a}+2\bar{a}\bar{\alpha }\right)}^{2}+4\bar{a}h\left(1+2\bar{\alpha }\right)\beta^{*}+4\beta^{*}{}^{2}\rho \sigma^{2}+8u_{0}}\right)}^{2}).$$

Specifically, we assume that ${\left(\bar{a}k+2\bar{a}k\bar{\alpha }\right)}^{2}-4k\left(\bar{\alpha }-\bar{a}h\beta^{*}-2\bar{a}h\bar{\alpha }\beta^{*}-\beta^{*}{}^{2}\rho \sigma^{2}-2u_{0}\right)>0$ always holds in our study. □

## Appendix F

**Proof of Lemma** **1.** Combining (A18) and (A19), the remanufacturer’s fixed payment under fairness is defined as

$$\alpha^{**}=\frac{1}{2}\left(\bar{\alpha }-{\bar{a}}^{2}k\bar{\alpha }-2{\bar{a}}^{2}k{\bar{\alpha }}^{2}-2\bar{a}h\bar{\alpha }\beta^{*}+\bar{a}\bar{\alpha }\sqrt{{\left(\bar{a}k+2\bar{a}k\bar{\alpha }\right)}^{2}-4k\left(\bar{\alpha }-\bar{a}h\beta^{*}-2\bar{a}h\bar{\alpha }\beta^{*}-\beta^{*}{}^{2}\rho \sigma^{2}-2u_{0}\right)}\right)$$

When $\alpha^{**}>\bar{\alpha }/2$, the inequality $\alpha^{**}-\bar{\alpha }/2>0$ holds, which implies

$$\frac{1}{2}(\bar{\alpha }-{\bar{a}}^{2}k\bar{\alpha }-2{\bar{a}}^{2}k{\bar{\alpha }}^{2}-2\bar{a}h\bar{\alpha }\beta^{*}$$

$$+\bar{a}\bar{\alpha }\sqrt{{\left(\bar{a}k+2\bar{a}k\bar{\alpha }\right)}^{2}-4k\left(\bar{\alpha }-\bar{a}h\beta^{*}-2\bar{a}h\bar{\alpha }\beta^{*}-\beta^{*}{}^{2}\rho \sigma^{2}-2u_{0}\right)})-\frac{1}{2}\bar{\alpha }$$

$$=\frac{1}{2}\bar{a}\bar{\alpha }(-\bar{a}k-2\bar{a}k\bar{\alpha }-2h\beta^{*}$$

$$+\sqrt{{\left(\bar{a}k+2\bar{a}k\bar{\alpha }\right)}^{2}-4k\left(\bar{\alpha }-\bar{a}h\beta^{*}-2\bar{a}h\bar{\alpha }\beta^{*}-\beta^{*}{}^{2}\rho \sigma^{2}-2u_{0}\right)})>0$$

Correspondingly, we have

$$\sqrt{{\left(\bar{a}k+2\bar{a}k\bar{\alpha }\right)}^{2}-4k\left(\bar{\alpha }-\bar{a}h\beta^{*}-2\bar{a}h\bar{\alpha }\beta^{*}-\beta^{*}{}^{2}\rho \sigma^{2}-2u_{0}\right)}>\bar{a}k+2\bar{a}k\bar{\alpha }+2h\beta^{*}.$$

Because both sides of the inequality are positive, both sides can be squared, and we get

$${\left(\bar{a}k+2\bar{a}k\bar{\alpha }\right)}^{2}-4k\left(\bar{\alpha }-\bar{a}h\beta^{*}-2\bar{a}h\bar{\alpha }\beta^{*}-\beta^{*}{}^{2}\rho \sigma^{2}-2u_{0}\right)>{\left(\bar{a}k+2\bar{a}k\bar{\alpha }+2h\beta^{*}\right)}^{2}.$$

After rearranging the inequality above, we derive the necessary and sufficient condition of $\alpha^{**}>\bar{\alpha }/2$, which expresses as

$$\alpha^{*}=\frac{1}{2}\left(-\frac{h^{2}\beta^{*}{}^{2}}{k}+\beta^{*}{}^{2}\rho \sigma^{2}+2u_{0}\right)>\bar{\alpha }/2.$$

Similarly, the necessary and sufficient condition of $\alpha^{**}<\bar{\alpha }/2$ is $\alpha^{*}<\bar{\alpha }/2$. □

## Appendix G

**Proof of Corollary** **2.** Combing (A18) and (A19), the collector’s effort level in the reciprocal model is defined as

$$a^{**}=\frac{2\alpha^{**}-\bar{\alpha }+2\bar{a}h\bar{\alpha }\beta^{*}}{2k\bar{a}\bar{\alpha }}=\frac{2\alpha^{**}-\bar{\alpha }}{2k\bar{a}\bar{\alpha }}+\frac{h\beta^{*}}{k}=\frac{2\alpha^{**}-\bar{\alpha }}{2k\bar{a}\bar{\alpha }}+a^{*}.$$

Therefore, when $\alpha^{**}>\bar{\alpha }/2$, $a^{**}>a^{*}$ holds. And because of τ=φ a, $\tau^{**}>\tau^{*}$ holds. Similarly, when $\alpha^{**}<\bar{\alpha }/2$, we get $a^{**}<a^{*}$ and $\tau^{**}<\tau^{*}$. □

## Appendix H

**Proof of Corollary** **3.** To compare the fixed payment α in rational and reciprocal models, we calculate the value of $\Delta \left(\alpha \right)=\alpha^{**}-\alpha^{*}$. It is derived that $\Delta \left(\alpha \right)=-\frac{k}{2}\left(a^{**}-a^{*}\right)\left(a^{**}+a^{*}+\bar{a}\right)$. When $\alpha^{**}>\bar{\alpha }/2$, $a^{**}>a^{*}$ holds (see Corollary 2.), which means $\Delta \left(\alpha \right)<0$. Therefore, we obtain that, when $\alpha^{**}>\bar{\alpha }/2$, $\alpha^{**}<\alpha^{*}$. Similarly, we also obtain that when $\alpha^{**}<\bar{\alpha }/2$, $\alpha^{**}>\alpha^{*}$. □

## Appendix I

**Proof of Corollary** **4.** From the equation

$$P_{r}{}^{**}-P_{r}{}^{*}=-\frac{-2b\phi -d\phi +2a^{**}bM\varphi -bdc_{m}-2b^{2}c_{r}}{4b^{2}-d^{2}}-\left(-\frac{-2bk\phi -dk\phi +2bhM\beta^{*}\varphi -bdkc_{m}-2b^{2}kc_{r}}{\left(4b^{2}-d^{2}\right)k}\right)=\frac{2bM\left(-a^{**}k+h\beta^{*}\right)\varphi }{\left(4b^{2}-d^{2}\right)k},$$

we get that when $\alpha^{**}>\bar{\alpha }/2$, $a^{**}>a^{*}=\frac{h\beta^{*}}{k}$ holds (See Corollary 3.), so we derive $P_{r}{}^{**}<P_{r}{}^{*}$; and when $\alpha^{**}<\bar{\alpha }/2$, $a^{**}<a^{*}=\frac{h\beta^{*}}{k}$, so we derive $P_{r}{}^{**}>P_{r}{}^{*}$. Similarly, from the equation

$$P_{m}{}^{**}-P_{m}{}^{*}=-\frac{-2b\phi -d\phi +a^{**}dM\varphi -2b^{2}c_{m}-bdc_{r}}{4b^{2}-d^{2}}-\left(-\frac{-2bk\phi -dk\phi +dhM\beta^{*}\varphi -2b^{2}kc_{m}-bdkc_{r}}{\left(4b^{2}-d^{2}\right)k}\right)=\frac{dM\left(-a^{**}k+h\beta^{*}\right)\varphi }{\left(4b^{2}-d^{2}\right)k},$$

we get that when $\alpha^{**}>\bar{\alpha }/2$, $a^{**}>a^{*}=\frac{h\beta^{*}}{k}$ holds, so we derive $P_{m}{}^{**}<P_{m}{}^{*}$; and when $\alpha^{**}<\bar{\alpha }/2$, $a^{**}<a^{*}=\frac{h\beta^{*}}{k}$ holds, so we derive $P_{m}{}^{**}>P_{m}{}^{*}$. □

## Appendix J

**Proof of Corollary** **5.** From the equation $S\left(\pi_{e}\right)=\alpha +\beta \pi_{e}$, we derive the total payoff S the remanufacturer pays to the collector in the rational model is $S^{*}=\alpha^{*}+h\;\beta^{*}a^{*}$, while in the reciprocal model, the total payoff is $S^{**}=\alpha^{**}+h\;\beta^{*}a^{**}$. To compare the total payoffs in rational and reciprocal models, we calculate the value of $\Delta \left(S\right)=S^{**}-S^{*}$. And we derive $\Delta \left(S\right)=\frac{k}{2}\left(a^{**}-a^{*}\right)\left(-a^{**}+a^{*}-\bar{a}\right)$. Here, $-a^{**}+a^{*}-\bar{a}\leq a^{*}-\bar{a}\leq 0$. Therefore, when $\alpha^{**}>\bar{\alpha }/2$, $a^{**}>a^{*}$ holds, and we have $S^{**}<S^{*}$. Similarly, when $\alpha^{**}<\bar{\alpha }/2$, $a^{**}<a^{*}$ holds, and we have $S^{**}>S^{*}$. □

## Appendix K

**Proof of Corollary** **6.** When $M^{2}<\frac{\left(4b^{2}-d^{2}\right)k\left(h^{2}+k\rho \sigma^{2}\right)}{2bh^{2}\varphi^{2}}$, $\frac{\partial a^{**}}{\partial \delta }=\frac{\left(4b^{2}-d^{2}\right)hkM\left(\bar{a}\left(h+2h\bar{\alpha }\right)+2\beta^{*}\rho \sigma^{2}\right)\varphi }{\left(4b^{2}-d^{2}\right)k\left(h^{2}+k\rho \sigma^{2}\right)-2bh^{2}M^{2}\varphi^{2}}>0$, $\frac{\partial \tau^{**}}{\partial \delta }>0$. And when $M^{2}>\frac{\left(4b^{2}-d^{2}\right)k\left(h^{2}+k\rho \sigma^{2}\right)}{2bh^{2}\varphi^{2}}$, $\frac{\partial a^{**}}{\partial \delta }<0$, $\frac{\partial \tau^{**}}{\partial \delta }<0$. □
